# Physical inactivity is associated with narrower lumbar intervertebral discs, high fat content of paraspinal muscles and low back pain and disability

**DOI:** 10.1186/s13075-015-0629-y

**Published:** 2015-05-07

**Authors:** Andrew J Teichtahl, Donna M Urquhart, Yuanyuan Wang, Anita E Wluka, Richard O’Sullivan, Graeme Jones, Flavia M Cicuttini

**Affiliations:** Department of Epidemiology and Preventive Medicine, School of Public Health and Preventive Medicine, Monash University, Alfred Hospital, 99 Commercial Road, Melbourne, VIC 3004 Australia; Baker IDI Heart and Diabetes Institute, 75 Commercial Road, Melbourne, VIC 3004 Australia; MRI Department, Healthcare Imaging Services, Epworth Hospital, 89 Bridge Road, Richmond, VIC 3121 Australia; Department of Medicine, Central Clinical School, Monash University, 89 Commercial Road, Melbourne, VIC 3004 Australia; Menzies Research Institute, 17 Liverpool Street, Hobart, TAS 7000 Australia

## Abstract

**Introduction:**

Although physical inactivity has been associated with numerous chronic musculoskeletal complaints, few studies have examined its associations with spinal structures. Moreover, previously reported associations between physical activity and low back pain are conflicting. This study examined the associations between physical inactivity and intervertebral disc height, paraspinal fat content and low back pain and disability.

**Methods:**

Seventy-two community-based volunteers not selected for low back pain underwent magnetic resonance imaging (MRI) of their lumbosacral spine (L1 to S1) between 2011 and 2012. Physical activity was assessed between 2005 and 2008 by questionnaire, while low back pain and disability were assessed by the Chronic Pain Grade Scale at the time of MRI. Intervertebral disc height and cross-sectional area and fat content of multifidus and erector spinae were assessed from MRI.

**Results:**

Lower physical activity levels were associated with a more narrow average intervertebral disc height (β −0.63 mm, 95% confidence interval (CI) −1.17 mm to −0.08 mm, *P* = 0.026) after adjusting for age, gender and body mass index (BMI). There were no significant associations between physical activity levels and the cross-sectional area of multifidus or erector spinae. Lower levels of physical activity were associated with an increased risk of high fat content in multifidus (odds ratio (OR) 2.7, 95% CI 1.1 to 6.7, *P* = 0.04) and high-intensity pain/disability (OR = 5.0, 95% CI 1.5 to 16.4, *P* = 0.008) after adjustment for age, gender and BMI.

**Conclusions:**

Physical inactivity is associated with narrower intervertebral discs, high fat content of the multifidus and high-intensity low back pain and disability in a dose-dependent manner among community-based adults. Longitudinal studies will help to determine the cause and effect nature of these associations.

**Electronic supplementary material:**

The online version of this article (doi:10.1186/s13075-015-0629-y) contains supplementary material, which is available to authorized users.

## Introduction

The Global Burden of Diseases study ranked low back pain the leading cause of disability of 291 conditions examined, and sixth in terms of disability-adjusted life years increasing from 58.2 million in 1990 to 83 million in 2010 [1]. Targeting modifiable risk factors associated with low back pain may therefore be helpful in reducing the burden of this disease.

Although physical inactivity has been associated with numerous chronic musculoskeletal complaints [2], two systematic reviews have shown the evidence linking physical activity and low back pain to be conflicting [3,4]. While one systematic review of 15 studies [3] found only one study demonstrating a significant positive relationship between physical inactivity and low back pain [5], the populations investigated were diverse, comprising both school children and adults. A more recent systematic review of 17 studies concluded that the relationship between physical activity and low back pain was too heterogeneous to reach any conclusion [4].

While studies have investigated, albeit inconclusively, the association between physical activity and back pain [3,4], the associations between physical activity and spinal structures have not been widely studied. Several studies have shown that mechanical stimulation is required to maintain intervertebral disc integrity [6,7], and that forced immobility is associated with muscle atrophy, such as a marked reduction in muscle size observed after spinal cord injury [8]. It may therefore be that physical inactivity plays a role in the pathogenesis of these structural abnormalities. Which structures are of clinical significance is, however, speculative, although intervertebral disc height and paraspinal muscle size and fat content have been shown to be associated with low back pain [9-11].

The aim of this study was to examine the associations between physical activity levels among community-based adults and 1) lumbar spine structures including intervertebral disc height and paraspinal muscle properties (cross-sectional area (CSA) and fat content), as well as 2) low back pain and disability. We hypothesised that physical inactivity would be independently associated with a narrow intervertebral disc, less CSA and high fat content of paraspinal muscles, as well as high-intensity low back pain and disability.

## Methods

### Participants

Seventy-two community-based individuals recruited through local media and weight loss clinics were examined as part of a study of obesity and musculoskeletal health [12]. Participants were recruited without reference to whether they had or did not have low back pain. Participants were excluded if there was a history of any arthropathy diagnosed by a medical practitioner (including inflammatory arthropathies or mechanical joint derangements), previous significant injury requiring non-weight-bearing therapy or prescribed analgesia, malignancy, contraindication to magnetic resonance imaging (MRI) or inability to understand English. The study was approved by the Human Research Ethics Committees of the Alfred Hospital and Monash University. All participants gave written informed consent.

### Physical activity level assessment

Physical activity levels for each participant were made available from a questionnaire conducted between 2005 and 2008 that has previously been used to assess exercise levels in healthy adults [13]. Participants were asked over the 14 days prior the questionnaire, how many days they had participated in strenuous activity for at least 20 minutes duration. Strenuous was defined as activity leading to sweating or shortness of breath and examples such as swimming, tennis, netball, athletics and running were listed. It has been shown that this intensity of exercise captured by questionnaire tends to better approximate habitual physical activity [14]. From these data, three categories of physical activity were extrapolated: active (9 to 14 days), moderately active (1 to 8 days) and inactive (0 days).

### Magnetic resonance imaging

MRI was performed (2011 to 2012) using a 3.0T magnetic resonance unit (MAGNETOM Verio, A Tim System; Siemens, Erlangen, Germany). The participant was positioned in the supine position and the following scans were performed: (1) sagittal T1 images from T12 to the sacrum (repetition time: 670 ms; echo time: 12 ms, slice thickness: 4 mm), (2) sagittal T2 images from T12 to sacrum (repetition time: 3,000 to 3,600 ms; echo time: 87 to 114 ms, slice thickness: 4 mm), and (3) axial T2 images from L1 to S1 (repetition time: 3,000 to 3,600 ms; echo time: 87 to 114 ms, slice thickness: 4 mm).

#### Intervertebral disc height

Intervertebral disc height of the lumbar spine was measured on mid-sagittal MR images from the middle of the superior border of the disc to the middle of the inferior border of the disc with the inclusion of both end plates. One trained observer measured the disc height in duplicate, one week apart, blinded to the characteristics of the participants. The intra-rater reliability of the disc height measures at each vertebral level was high, with intra-class correlation coefficients (ICCs) ranging from 0.94 to 0.98. The average disc height was calculated by summing L1/2, L2/3, L3/4, L4/5, and L5/S1 intervertebral disc height and dividing by five.

#### Muscle cross-sectional area and fat content

Paraspinal muscle CSA and fat content were determined at the level of the L3/L4 intervertebral disc. The CSA of multifidus and erector spinae were measured by outlining the border of each muscle using a tool in OsiriX. The measurements were repeated by the same observer, two weeks apart. To avoid bias, the observer was blinded to the first measurement when performing the second measurement. The intra-observer reliability (ICC) was 0.99.

**Figure 1:**
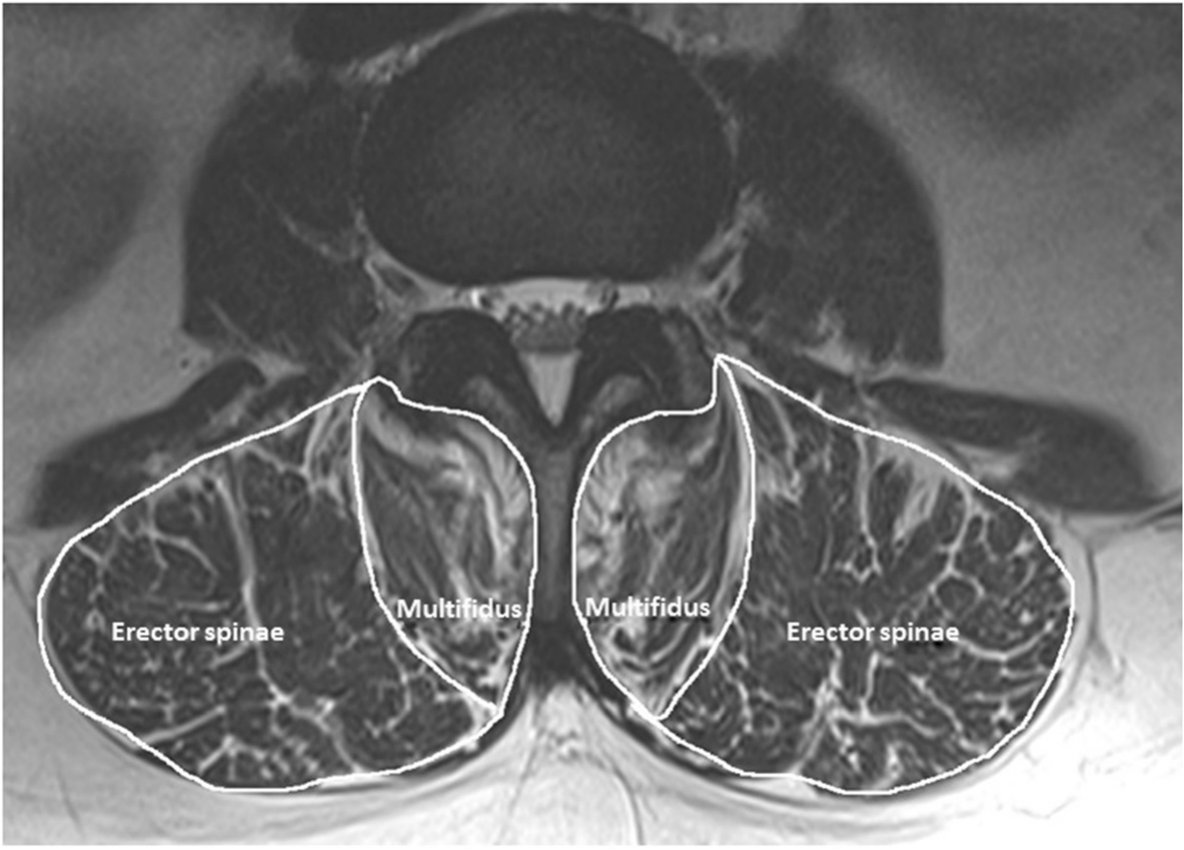
An example of high fat (>50%) content of multifidus but not erector spinae.

Hyperintense regions within the paraspinal muscles observed on T2 axial images were considered fatty tissue [15] and categorised based on a previously validated grading method; grade 0: no fat, grade 1: 1 to 10% fat, grade 2: 11 to 50% fat and grade 3: >50% fat [15]. The intra-observer reliability (ICC) for the paraspinal muscle fat content for both multifidus and erector spinae was 0.99. High fat content was defined as greater than 50% of the muscle (see Figure 1).

### Anthropometric data

Height was measured to the nearest 0.1 cm using a stadiometer. Weight was measured to the nearest 0.1 kg using a single pair of electronic scales. Body mass index (BMI) (kg m^−2^) was calculated.

### Chronic pain and disability

The Chronic Pain Grade Questionnaire was administered at the time of MRI in 2011 to 2012 to obtain information on low back pain intensity over the past six months. The Chronic Pain Grade Questionnaire is a reliable and valid instrument for use in population surveys of low back pain [16,17]. The questionnaire includes seven questions from which a pain intensity and disability subscale score are calculated. Subscale scores for pain intensity and disability are combined to calculate a chronic pain grade that enables classification of chronic pain into five hierarchical categories: grades 0 (no pain) to 4 (high disability, severely limiting) as previously described [16,17]. High-intensity pain/disability was defined as being of either grade 2 (low disability but high intensity), grade 3 (high disability, moderately limiting) or grade 4 (high disability, severely limiting).

### Statistical analyses

The inactive, moderately active and active groups were compared. The F test was used to determine the pairwise comparison among the estimated marginal means for continuous data, chi-squared tests for categorical data and the Fisher’s exact test for chronic pain grade data. Multiple linear regression analyses were used to examine the associations between physical activity levels and intervertebral disc height, adjusted for age, gender and BMI. Multiple linear regression analyses were also employed to examine the relationship between physical activity levels and paraspinal muscle CSA, adjusted for age, gender, BMI and fat content. Binary logistic regression analyses were used to examine the relationships between physical activity levels and the risk of high (>50%) paraspinal muscle fat content and high-intensity pain/disability, with adjustment made for age, gender and BMI. We chose to examine muscle variables on the left side only for standardisation purposes and to avoid multiple testing. A *P* value of less than 0.05 (two-tailed) was regarded as statistically significant. All analyses were performed using the IBM SPSS statistical package (standard version 20.0 IBM Corp., Armonk, NY, USA).

## Results

The majority of participants in the study were female (68.1%), and the average BMI was in the overweight category (29.2 kgm^−2^). Approximately 21% of the participants were inactive, and 53% and 26% had moderate and high levels of physical activity respectively.

Participant characteristics (N = 72) are shown in Table 1, according to their activity levels. People with higher activity levels had a smaller weight (*P* = 0.02) and BMI (*P* = 0.002) than their less physically active counterparts. The average intervertebral disc height in the lumbosacral (L1 to S1) spine was narrower the more physically inactive a person was (inactive: 10.2 (standard error of the mean (SEM) 4.1) mm, moderately active 10.6 (SEM 2.6) mm and active 11.8 (SEM 3.6) mm, *P* = 0.01 for difference among the three groups). High fat content of multifidus was more prevalent in the less physically active groups (inactive: 60%, moderately active: 34.2%, active: 15.8%; *P* = 0.03). The chronic pain grade was greater in the less physically active groups (*P* = 0.049).

**Table 1:** **Participant characteristics**

	**Inactive**	**Moderately active**	**Active**	***P***
	**(0 days)**	**(1 to 8 days)**	**(9 to 14 days)**	
	**N = 15**	**N = 38**	**N = 19**	
**Age (years)** ^*^	47.2 (1.3)	45.1 (0.7)	46.5 (1.5)	0.29
**Gender (n, % female)** ^**^	13 (86.7)	26 (68.4)	10 (52.6)	0.11
**Weight (kg)** ^*^	99.0 (3.6)	96.5 (2.1)	84.4 (4.3)	0.02
**Height (m)** ^*^	1.67 (0.01)	1.67 (0.01)	1.70 (0.02)	0.21
**BMI (kgm** ^**−2**^ **)** ^*^	35.7 (1.3)	34.8 (0.8)	29.3 (1.5)	0.002
**Intervertebral disc height (L1 to S1) (mm)** ^*^				
*Average*	10.2 (4.1)	10.6 (2.6)	11.8 (3.6)	0.01
**Paraspinal muscle properties**				
**CSA (cm** ^**2**^ **)** ^*^				
*Multifidus*	6.9 (0.4)	6.9 (0.3)	7.2 (0.4)	0.76
*Erector spinae*	19.7 (1.1)	20.5 (0.7)	21.7 (1.0)	0.41
**High fat content, n (%)** ^**^				
*Multifidus*	9 (60.0)	13 (34.2)	3 (15.8)	0.03
*Erector spinae*	5 (33.3)	7 (18.4)	1 (5.3)	0.11
**Chronic low back pain grade, n (%)** ^***^				
*Pain free*	3 (20.0)	6 (15.8)	5 (26.3)	0.049
*Low disability, low intensity*	5 (33.3)	24 (63.2)	14 (73.7)	
*Low disability, high intensity*	4 (26.7)	1 (2.6)	0 (0.0)	
*High disability, moderately limiting*	1 (6.7)	4 (10.5)	0 (0.0)	
*High disability, severely limiting*	2 (13.3)	3 (7.9)	0 (0.0)	
**High pain/disability, n (%)** ^***^	7 (46.7)	8 (21.1)	0 (0.0)	0.004

Results displayed as mean (standard error of the mean) unless otherwise stated. ^*^ *P* values determined from pairwise comparison among the estimated marginal means; ^**^ *P* values determined from chi-squared test; ^***^ *P* values determined from Fisher’s exact test. BMI, body mass index; CSA, cross-sectional area.

Lower physical activity level was associated with an increased risk of high-intensity pain/disability (odds ratio (OR) = 5.0, 95% confidence interval (CI) 1.5 to 16.4, *P* = 0.008) after adjustment for age, gender and BMI (data not shown). Moreover, in univariate analyses, lower physical activity levels were associated with more narrow average lumbosacral intervertebral disc height (β −0.78 mm, 95% CI −1.31 mm to −0.23 mm, *P* = 0.006). This association persisted after adjusting for age, gender and BMI (β −0.63 mm, 95% CI −1.17 mm to −0.08 mm, *P* = 0.026).

The associations between activity levels and paraspinal muscle properties are shown in Table 2. There were no significant associations between physical activity levels and the CSA of multifidus or erector spinae. In univariate analyses, lower levels of physical activity were associated with an increased risk for high fat content in multifidus (OR 2.8, 95% CI 1.3 to 6.3, *P* = 0.01). This relationship persisted after adjusting for age, gender and BMI (OR 2.7, 95% CI 1.1 to 6.7, *P* = 0.04). In univariate analyses, lower levels of physical activity were associated with an increased risk for high fat content in erector spinae (OR 2.7, 95% CI 1.0 to 7.1, *P* = 0.04). This relationship failed to maintain statistical significance after adjusting for age, gender and BMI (OR 2.2, 95% CI 0.7 to 6.9, *P* = 0.16). When the low back pain grade was added to the multivariate analyses, results were unchanged (data not shown).

**Table 2:** **The associations between lower levels of physical activity and paraspinal muscle properties**

**Risk of high fat content**				
	**Univariate**	***P***	**Multivariate**	***P***
	**OR (95% CI)**		**OR (95% CI)** ^**1**^	
*Multifidus*	2.8 (1.3, 6.3)	0.01	2.7 (1.1, 6.7)	0.04
*Erector spinae*	2.7 (1.0, 7.1)	0.04	2.2 (0.7, 6.9)	0.16
**Cross-sectional area (cm** ^**2**^ **)**				
	**Univariate**	***P***	**Multivariate**	***P***
	**β (95% CI)**		**β (95% CI)** ^**2**^	
*Multifidus*	0.15 (−0.43, 0.73)	0.61	0.09 (−0.48, 0.66)	0.76
*Erector spinae*	−1.01 (−2.53, 0.50)	0.19	−0.58 (−2.07, 0.91)	0.44

^1^Risk of fat infiltration adjusted for age, gender, and BMI; ^2^cross-sectional area adjusted for age, gender, BMI and fat content. OR, odds ratio; CI, confidence interval; BMI, body mass index.

## Discussion

This study has demonstrated a dose-response relationship between physical inactivity and structural abnormalities in the lumbosacral spine, including narrow intervertebral disc height and an increased risk for high (>50%) multifidus fat content. Moreover, there was a dose-response relationship between physical inactivity and the risk for high-intensity low back pain/disability. These data provide the first evidence that among community-based adults, physical inactivity is associated with deleterious changes in the structure of the lumbosacral spine.

Intervertebral disc narrowing is a feature of degenerative disc disease, suggested by some to be the single most important structural risk factor for low back pain [18]. Nevertheless, degenerative disc disease is an umbrella term and classification systems vary across studies [19]. Since lumbar intervertebral disc height has been associated with low back pain [11], it is likely to represent a clinically important structural endpoint that is not reliant on a whole host of other radiological features, such as signal change in the intervertebral disc. In this study, we have demonstrated a dose-response association between physical activity and the average intervertebral disc height in the lumbosacral spine. Moreover, in this study, disc height has been assessed quantitatively and treated as a continuous variable, providing a more sensitive assessment than previous methods that have employed qualitative descriptors such as ‘normal to slightly decreased’ intervertebral disc height as a measure of disc degeneration [20]. Why low levels of physical activity may be associated with a reduction in intervertebral disc height is speculative. One possibility is that low levels of physical activity may result in inadequate mechanical stimulation, a factor important in maintaining intervertebral disc integrity [6,7]. It is also possible that a narrower intervertebral disc may cause pain and resultant low levels of physical activity. However, when we adjusted for the low back pain grade, results were unchanged (data not shown), suggesting that low levels of physical activity are associated with more narrow intervertebral disc height, independent of pain and disability.

Forced immobility has previously been shown to be associated with muscle atrophy, such as a marked reduction in muscle size observed after spinal cord injury [8]. Such data lead us to hypothesise that physical inactivity would be associated with less paraspinal muscle CSA. Nevertheless, this study failed to observe any associations between physical activity and the CSA of paraspinal muscles. Fat infiltration is a sign of muscle atrophy [9,10] that may occur prior to any reduction in a muscle CSA. For instance, replacement of muscle with fat, while changing the function of the muscle, may not significantly alter its CSA [21]. No previous study has examined the association between physical activity levels and fat replacement of paraspinal muscles. Histological studies have demonstrated concordance between intermuscular adipose tissue detected by MRI and intra-operative specimens of paraspinal muscles [22], with other studies corroborating MRI as a valid method of identifying the amount of fat in the skeletal muscles [23,24]. Severe fat infiltration of multifidus has strongly been associated with clinical endpoints, such as ever having had low back pain or low back pain in the last year [9]. Using proton MR spectroscopy, a previous study found that compared to 25 asymptomatic controls, participants with chronic low back pain had a significantly higher fat content in multifidus [10]. The current study has demonstrated that independent of age, gender and BMI, low levels of physical activity were associated high fat content of multifidus. Adjusting for the chronic pain grade did not significantly alter the results of the study. Therefore, in a population not selected on the basis of chronic back pain, physical inactivity may be associated with higher fat content, rather than a change in the CSA of multifidus. A similar direction of results was observed between physical inactivity and a high fat content of erector spinae, although this did not reach statistical significance. This may reflect the modest sample size and larger cohorts may help to confirm or refute this relationship.

Finally, this study demonstrated that lower levels of physical activity were associated with an increased risk for high-intensity low back pain and disability in community-based adults. A recent systematic review of 17 studies concluded that the relationship between physical activity and low back pain was too heterogeneous to reach any conclusion [4]. However, on closer scrutiny of this systematic review [4], all adult studies reported significant associations between low physical activity and an increased risk of low back pain [25-27], while inconsistent relationships were more common in studies examining school children [28-33]. Our results provide further support that in an adult population, physical inactivity is associated with high-intensity low back pain and disability.

This study was limited by its modest sample size and predominance of female participants (68.1%), so larger studies incorporating more male participants are required to substantiate the generalisability of our findings. Moreover, this study cannot determine causality. That is, whether low levels of physical activity are the cause or result of a narrow disc, high multifidus fat content or low back pain/disability cannot be determined from these data. Longitudinal studies will help to clarify such issues. However, it is more biologically plausible that a narrow intervertebral disc, high fat content of multifidus or low back pain/disability were the result of physical inactivity, rather than vice versa. Indeed, physical activity was measured between 2005 and 2008, which preceded the MRI spinal structure and low back pain assessment that was performed between 2011 and 2012. It has been shown that this intensity of exercise captured by questionnaire tends to better approximate habitual physical activity levels [14]. Based on validated methods, we employed a semi-quantitative method for assessing fat replacement of paraspinal muscles [9,15,34] and assessed not only low back pain, but a composite score of pain and disability [16,17]. Although we acknowledge the potential for these methods to result in misclassification, this non-differential misclassification would have reduced this study’s ability to show any significant associations. Additionally, the Chronic Pain Grade Questionnaire provides a composite score of pain and disability, but does not characterise pain (for example radicular and/or mechanical pain). Moreover, our means of assessing activity levels were derived from asking subjects the number of days in the fortnight they had participated in strenuous activities that had resulted in them sweating or experiencing shortness of breath for at least 20 minutes duration. A quantitative measure of physical activity such as measurement via an accelerometer may help to substantiate the findings in this study. This study had MRI scans performed sporadically throughout the day in an attempt to mitigate any diurnal variation that may influence intervertebral disc height. Any diurnal pattern of intervertebral disc height variability would have once again introduced non-differential misclassification into the study, reducing the chances of finding statistically significant results. While we have selected a community-based population, this study had a selection bias towards overweight and obese adults. This may limit the generalisability of our findings to those of normal weight. Finally, we assessed muscle CSA and fat replacement at the level of L3/4 disc level on the left side. This anatomical region was chosen as it approximated a mid-point of the lumbar spine. Analyses at each individual level would have introduced the issue of multiple testing and was therefore avoided. Nevertheless, it is possible that physical activity may have differential effects on paraspinal muscle properties at different spinal levels. The left side was arbitrarily chosen as we presumed that it may have greater fat content, being the non-dominant side in most individuals. Nevertheless, the correlation between muscle CSA between the left and right sides for multifidus and erector spinae was strong (r = 0.87 and 0.92, respectively). Similarly, the correlation between the left and right sides for multifidus and erector spinae fat infiltration was also strong (r = 0.82 and 0.95, respectively). We performed *post hoc* analyses and demonstrated similar associations between physical activity and right-sided paraspinal muscle properties (data not shown).

## Conclusions

This study of community-based adults demonstrated a dose-response relationship between physical inactivity and a narrow intervertebral disc as well as high multifidus fat content. We also found that physical inactivity was associated with high-intensity pain and disability. Longitudinal studies are required to determine cause and effect between these associations.

## Abbreviations

BMI: body mass index
CI: confidence interval
CSA: cross-sectional area
ICC: intra-class correlation coefficient
MRI: magnetic resonance imaging
OR: odds ratio
SEM: standard error of the mean
